# Comparative Analysis of Kinetic Parameters of Sustainable Branched Esters Obtained from Lauric Acid

**DOI:** 10.1021/acsomega.5c10304

**Published:** 2026-02-03

**Authors:** María Gómez, María Dolores Murcia, Elisa Gómez, Asunción Hidalgo, Fuensanta Máximo, María Claudia Montiel

**Affiliations:** Chemical Engineering Department, 16751 University of Murcia, Campus de Espinardo, 30071 Murcia, Spain

## Abstract

A comparison between four esterification reaction systems to obtain new sustainable branched esters using Novozym 435 as a biocatalyst, the same acid (lauric acid), and four alcohols with different chain lengths and side chains (2-hexyl-1-decanol, 2-ethyl-1-hexanol, 2-butyl-1-octanol, and 3,7-dimethyl-1-octanol) has been carried out. The parameters of the reaction have been optimized in 0.5 g of biocatalyst, temperature of 70 °C, and the stoichiometric molar ratio (1:1). Under these conditions, conversion values of >90% are obtained in the four reactions. Using a kinetic model developed by the authors and based on a Bisubstrate Ping-Pong mechanism, where internal diffusional limitations are considered, the kinetic parameters for each reaction system were determined and the theoretical conversion values closely matched the experimental results, validating the model for this wide range of substrates. Attending at the conversion values obtained, where both the reaction rate and transport rate are considered, the esterification with 3,7-dimethyl-1-octanol leads to the highest average rate, followed by the reactions with 2-ethyl-1-hexanol, 2-butyl-1-octanol, and, finally, 2-hexyl-1-decanol. In the first two systems, the ones with alcohols of shorter side chain and chain length, respectively, the *k*_cat_ values are very high (49.526 and 90.13 Mh^–1^ g^–1^, respectively) and so is the reaction rate, leading to a high average rate. However, when 3,7-dimethyl-1-octanol is used, the conversion values decrease at long reaction times, due to the high volatility of this alcohol. In the reaction system with 2-butyl-1-octanol, there is mixed control of the reaction and transport stages with higher values of the effectiveness factor (above 0.5 in most cases). Finally, in the reaction with 2-hexyl-1-decanol, the alcohol with the longest chain length and side chain, and the highest molecular weight and viscosity, internal diffusional limitations are very high (with low values of the effectiveness factors as expected, around 0.2 for all conditions tested), and the reaction rate is quite low as well, which explains the low average rates obtained. The obtained branched esters are of interest in the biolubricant sector, and the kinetic parameters calculated in this study can be useful to allow simulation, further optimization, and scale up of the esterification process.

## Graphical abstract

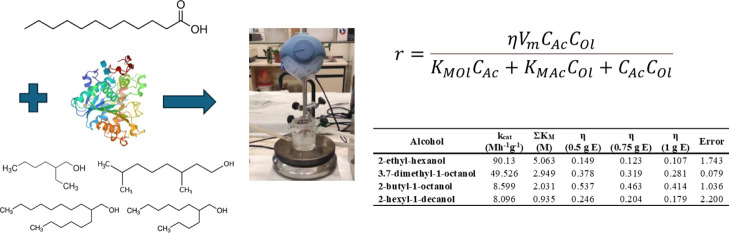

## Introduction

Enzymatically synthesized esters have gained significant industrial relevance due to their high selectivity, mild reaction conditions, and reduced environmental impact compared to conventional chemical routes. These biocatalytically produced compounds are widely applied in sectors such as food flavoring, fine chemicals, pharmaceuticals, and biodegradable lubricants, where product purity and stereochemical control are critical.

Branched esters have lower melting points than straight-chain esters due to reduced molecular packing, resulting in weaker intermolecular attractions. These lower melting points are of interest in many uses. Ester applications include serving as solvents for resins, contributing to artificial flavors and fragrances, and being of significant interest in three major industries: biolubricants,^1^ biodiesel,^2^ and cosmetics.^3^ In lubricants, the base oil is a crucial component, influencing the lubricant properties and durability. Three main types of lubricants exist: mineral (from petroleum fractions), natural (from sources like animal fats or vegetable oils), and synthetic (using compounds such as polyalphaolefins and esters produced through chemical synthesis). In recent years, significant efforts have been dedicated to exploring alternatives to conventional lubricants.^4^ Researchers have investigated various esterification and transesterification reactions catalyzed by enzymes to produce branched esters that can be used as biolubricants. Examples include the synthesis of compounds like bis­(2-ethylhexyl) adipate,^5^ dilauryl adipate,^6^ dimethyl adipate,^7^ dioctyl adipate,^8^ bis­(2-ethylhexyl) sebacate, bis­(3,5,5-trimethylhexyl) sebacate,^9^ dioctyl sebacate,^10^ azelaic acid-derived esters,^11,12^ bis­(2-ethylbutyl) adipate, and bis­(2-ethylbutyl) sebacate.^13^ The biocatalysts Novozym 435 and Lipozyme are among the most commonly employed enzymes for these processes.

Enzymes are efficient biocatalysts with specific properties, but their biological origins and regulation make their industrial use challenging. Modifications, including enzyme immobilization, are necessary to achieve the desired stability. Lipases, commonly used in biocatalysis, have broad selectivity and are highly stable. They employ an “interfacial activation” mechanism, involving a hydrophobic pocket covered by a lid. This lid can move to expose the active center when interacting with a hydrophobic substrate, shifting the enzyme into an active state. This mechanism allows lipases to function effectively in various reactions, including esterification.^1^ When it comes to the positioning of enzymes within the reaction medium, immobilized enzymes have certain advantages over free enzymes in suspension. They can be easily separated in the final purification process and can be reused in subsequent experiments.^14^ However, in these scenarios, the reaction takes place within the catalytic particles. As a result, the synthesis process can be influenced by mass transfer processes, including internal and external diffusion of substrates and products.^15^ Lately, there has been increasing interest in developing enzymatic processes in solvent free systems, as they align with the principles of Green Chemistry, reduce toxic waste, simplify downstream purification processes, and lower the overall operational costs. These systems have thermodynamic and kinetics implications on reaction that must be taken into account.^16^

There is a lack of detailed reaction mechanisms and kinetic equations available for some processes involving branched esters, and researchers have sought to optimize experimental conditions using methods like Surface Response Methodology.^6,12,17,18^ This highlights the complexity and specificity of certain esterification reactions, particularly those catalyzed by lipases, in which monocarboxylic acids and monohydroxylic alcohols are used to obtain esters. In some cases, the kinetics of these reactions are described by a bisubstrate ping-pong equation.^19–22^ The bisubstrate ping-pong kinetics involve multiple reaction steps and are characterized by substrates binding to and releasing from the enzyme (catalyst) in a sequential manner. To determine the kinetic parameters for such reactions, some authors have resorted to solving nonlinear differential equations associated with the ping-pong model using numerical calculation methods.^23,24^ This approach allows for a more comprehensive understanding of the kinetics of these reactions as it considers the complexities of the catalytic mechanism. The use of numerical calculation and modeling techniques can be valuable in gaining insights into these complex kinetic processes and optimizing the reaction conditions for the production of branched esters.

A software tool based on Visual Basic for Applications (VBA)^16^ was utilized to investigate the kinetics of the synthesis of bis­(2-ethylhexyl) azelate using Novozym 435 in a solvent-free system. Building on this experience, a similar approach was employed to study the synthesis of laurate of 2-hexyl-1-decanol,^25^ where the kinetic parameters were calculated using the Excel Solver tool. Once confirmed that the tool works, in this study, it has been used to calculate the kinetic parameters of four esters formed from the transesterification reaction of lauric acid with four alcohols with different carbon-chain lengths and side-chain structures. Internal diffusional limitations have been considered, reaction and transport rates for each reaction have been compared, and the kinetic parameters obtained, with the aim of confirming the consistency of the proposed kinetic model for a wider range of substrates and studying the influence of their different structures and properties in the obtained results.

## Material and Methods

### Reagents

Lauric acid (99%) was purchased from Acros Organics, 2-hexyl-1-decanol (97%), 2-ethyl-1-hexanol (99%), 2-butyl-1-octanol (95%), 3,7-dimethyl-1-octanol (98%), and methyl myristate (99%) were purchased from Sigma-Aldrich, and *n*-heptane (99%) was purchased from PanReac AppliChem.

The lipase-based biocatalyst, Novozym 435 (Candida antarctica lipase B immobilized on a macroporous acrylic resin, 10,000 PLU/g, 1 PLU is the amount of enzyme activity which generates 1 μmol of propyl laurate per minute under defined standard conditions) was kindly provided by Novozymes Spain S.A.

Methyl myristate (≥99%) from Sigma-Aldrich was used as an internal standard for the gas chromatography analysis of the samples. Other reagents and products were of analytical grade.

Table 1 depicts the physical properties of the four alcohols.

**Table 1:** Physical Properties of Alcohols

alcohol	molecular weight (g mol^–1^)	density (g cm^–3^)70 °C	viscosity (mPa.s)70 °C	boiling point (°C)
**2-ethyl-hexanol**	130.23	0.79505	1.766	183–186
**3,7-dimethyl-1-octanol**	158.28	0.7934	2.675	98–99
**2-butyl-1-octanol**	186.33	0.79927	3.401	145–149
**2-hexyl-1-decanol**	242.44	0.783636	5.468	193–197

### Experimental Method

The experimental procedure was the same for each reaction system tested. An open-air jacketed stirred batch reactor of 50 cm^3^ total volume was used to carry out the reactions. A stirring speed of 300 rpm, previously optimized to avoid external diffusion limitations, was kept constant for all assays. The reactions took place in a solvent-free medium, where the total mass of acid plus alcohol was 20 g. Lauric acid was added first due to its solid state. When it is melting, the corresponding alcohol is added to the reactor. Finally, when the required temperature is reached, the enzyme is introduced.

The reaction progress was followed by taking samples of 10 μL which were diluted in heptane and analyzing the concentrations of residual substrates and products until a reaction time of 300 min.

All the experiments were run in duplicate, and the maximum calculated standard deviation using the RegressIt Excel tool was 3.1%.

### Analytical Method

The analytical approach employed represents an adaptation of a previously established method within our research team.^26^ In this study, the analysis of substrates and products was conducted utilizing an Agilent 7820A Gas Chromatograph (GC), equipped with a Flame Ionization Detector (FID) and a silica capillary column measuring 30 m in length, 0.32 mm in diameter, and 0.25 μm in film thickness.

The specific analytical conditions were as follows: The injector temperature was set at 250 °C, the detector temperature was maintained at 300 °C, and a 2:1 split ratio was employed. Nitrogen was utilized as the carrier gas, flowing at a rate of 1 mL per minute. The oven temperature was held steady at 80 °C for 1 min and then ramped up to 120 °C at a rate of 75 °C per minute. After an additional minute, it was further increased to 290 °C at a rate of 20 °C/min, which was maintained for 3.5 min.

Injections consisted of 1 μL of diluted samples, and the entire analysis process took approximately 14.53 min to complete. Methyl myristate served as the internal standard in the determination of both substrates, with the final product concentration determined by the total mass balance. The use of an internal standard ensures the reliability of the analytical method employed.

The obtained calibration curves for the different substrates, where *y* = [concentration] (mM) and *x* = substrate area/methyl myristate area, are the following ones:

2-ethyl hexanol: *y* = 42.845*x*.

3,7-dimethyl-1-octanol: *y* = 74.038*x*.

2-butyl-1-octanol: *y* = 51.294*x*.

2-hexyl-1-decanol *y* = 22.134*x*.

Lauric acid: *y* = 38.447*x*.

### Experimental Series

Three experimental series were carried out for each reaction system: biocatalyst amount variation, temperature variation, and molar ratio acid:alcohol variation. In the first series, the different biocatalyst amounts tested were 0.5, 0.75, and 1 g, keeping constant a temperature of 70 °C and a molar ratio 1:1 (the stoichiometric one). In the second series, three temperature values were tested, 65, 70, and 75 °C, at a fixed biocatalyst amount of 0.5 g and molar ratio 1:1. And finally, in the last series, biocatalyst amount and temperature were kept constant at 0.5 g and 70 °C, respectively, and the molar ratio acid:alcohol was varied at 1:1, 1.25:1, and 1.5:1.

## Results and Discussion

### Theory

The kinetic model that has been used to fit all the experimental data was developed by the authors and explained in detail in a previous work.^25^ The model can be applied to esterification reactions where immobilized enzymes are used as catalysts, so that the kinetic type is a bisubstrate Ping-Pong with internal diffusional limitations:

1 $$r=\frac{\eta V_{m}C_{Ac}C_{Ol}}{K_{MOl}C_{Ac}+K_{MAc}C_{Ol}+{\;C}_{Ac}C_{Ol}}$$

Once the alcohol (limiting substrate) mass balance applied to a batch reactor is done, substituting eq 1 in the mass balance, applying the conversion definition and integrating the resulting equation as explained in the literature,^25^ an equation for the reaction time as a function of the conversion and the kinetic parameters is obtained:

2 $$t=\frac{C_{Ol0}X-{\;K}_{MOl}\mathrm{Ln}\left(1-X\right)-K_{MAc}\mathrm{Ln}\left(1-\frac{C_{Ol0}}{C_{Ac0}}X\right)}{\eta V_{m}}$$

In particular, when the initial concentrations of the acid and alcohol are equal, the previous equation simplifies to

3 $$t=\frac{C_{Ol0}X-\left(\sum K_{M}\right)\;\mathrm{Ln}\left(1-X\right)}{\eta V_{m}}$$

All of the kinetic parameters used are detailed in the Nomenclature section. The effectiveness factor is the one corresponding to a first order kinetics, since, as previously explained,^16^ there is no significant difference with the one obtained for a Michaelis–Menten kinetics type.

For each reaction system, all experimental data have been fitted to the proposed kinetic model, and the methodology used to obtain the kinetic parameters was as follows:

The Excel Solver tool has been used to determine the unknown parameters of the previous equation and the theoretical reaction times using, as convergence condition, the minimum value of the sum of square error between the calculated time and the experimental one:

4 $$Sum\;error=\sum_{0}^{t}\;{\left(t-t_{calc}\right)}^{2}$$

In addition, once the kinetic parameters are obtained, using the bisection method implemented in a Visual Basic program developed by the authors, the theoretical values of the conversion have been calculated and compared with the experimental values for each assay. The results obtained for each series and for the different reaction systems are shown below.

### Results for the Biocatalyst Variation Series

Figure 1 shows the results obtained with each reaction system (same acid, different alcohols) for all the biocatalyst amounts tested, the dots corresponding to the experimental conversion values, and the continuous lines to the values calculated with the proposed kinetic model.

**Figure 1:**
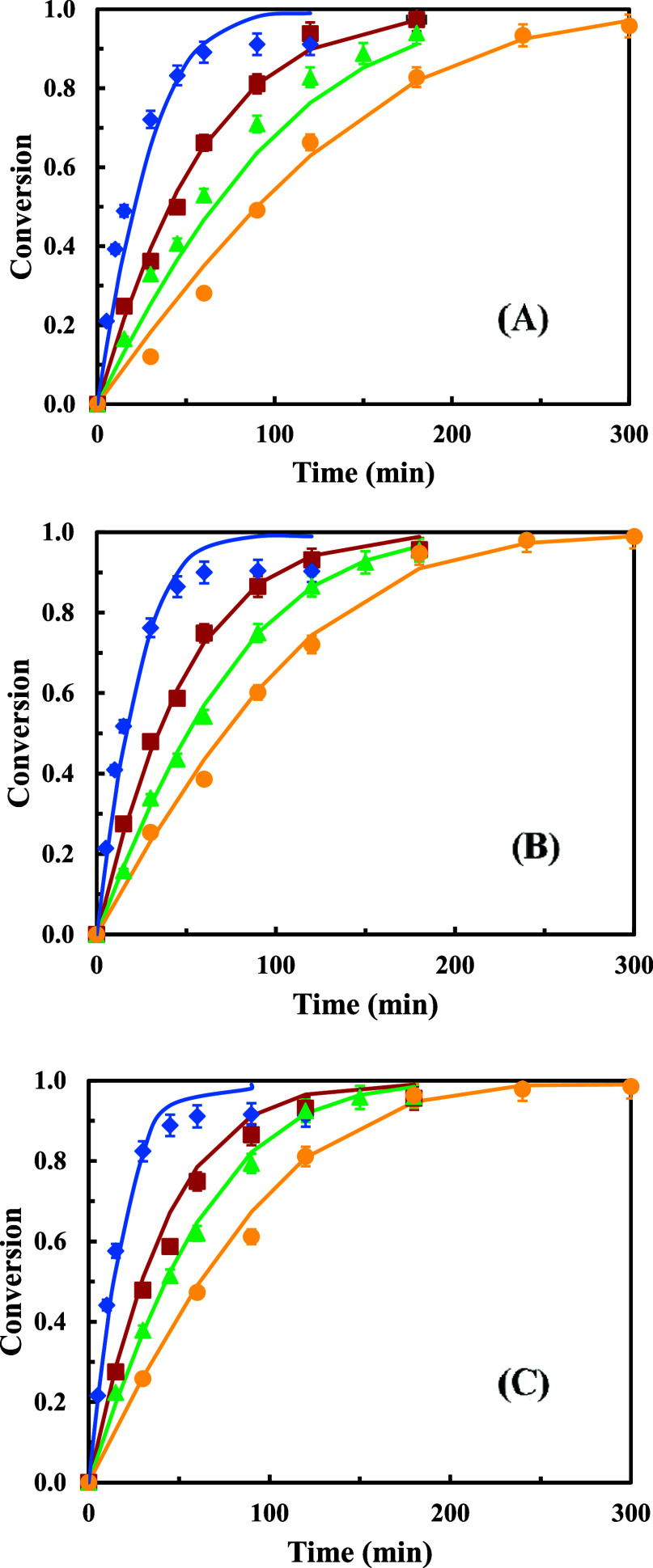
Experimental and calculated conversion values versus time for each reaction system and lauric acid with different alcohols using Novozym 435 as a biocatalyst: (●) 2-hexyl-1-decanol, (▲) 2-butyl-1-octanol, (■) 2-ethyl-1-hexanol, and (⧫) 3,7-dimethyl-1-octanol, (−) calculated. Amount of biocatalyst: (A) 0.5 g, (B) 0.75 g, and (C) 1.0 g. Temperature of 70 °C and a molar ratio acid:alcohol 1:1.

As depicted in Figure 1, the same trend was obtained for all the biocatalyst amounts: the maximum conversion values up to 60–90 min reaction time are reached when the alcohol 3,7-dimethyl-1-octanol is used, followed by 2-ethyl-1-hexanol, 2-butyl-1-octanol, and, finally, 2-hexyl-1-decanol. Also, as expected, the reaction is faster when using higher biocatalyst amounts. However, 0.5 g of biocatalyst (Figure 1A) has been selected as optimum, since it allows reaching high conversion values, and although it requires a bit longer reaction time, the savings in biocatalyst costs compensates for this.

In the case of the esterification with 3,7-dimethyl-1-octanol, from approximate 1 h, the conversions are constant, due to the higher volatility of this alcohol, which is partially lost, causing the reaction to stop. This is why at long reaction times, the experimental conversion values obtained with this alcohol are a bit lower compared with the theoretical conversion values and with those obtained with the other alcohols.

As can be seen in Figure 1, there is a high degree of agreement between experimental conversions and the ones calculated using the proposed kinetic model, for all biocatalyst amounts tested and all the reaction systems, with the exception, previously commented, of the system with 3,7-dimethyl-1-octanol at longer reaction times.

The kinetic parameters calculated for this series are shown in Table 2, as well as the error defined in eq 4. In order to explain the obtained results, the definition of the effectiveness factor in heterogeneous reactions with enzymes immobilized on porous supports must be considered:

5 $$\eta =\left(\frac{Average\;reaction\;rate}{reaction\;rate\;without\;internal\;difussional\;limitations}\right)$$

**Table 2:** Kinetic Parameters Obtained for the Series of Biocatalyst Amount Variation

alcohol	*k*_cat_ (Mh^–1^ g^–1^)	Σ*K*_M_ (M)	η (0.5 g E)	η (0.75 g E)	η (1 g E)	error
**2-ethyl-hexanol**	90.13	5.063	0.149	0.123	0.107	1.743
**3.7-dimethyl-1-octanol**	49.526	2.949	0.378	0.319	0.281	0.079
**2-butyl-1-octanol**	8.599	2.031	0.537	0.463	0.414	1.036
**2-hexyl-1-decanol**	8.096	0.935	0.246	0.204	0.179	2.200

In the case of the reaction system with 3,7-dimethyl-1-octanol, the value of *k*_cat_ is quite high, 49.526 Mh^–1^ g^–1^, so the reaction stage is fast, this involves a high control of the internal transport and a low value of the effectiveness factor (0.378, 0.319, and 0.281 for 0.5, 0.75, and 1 g biocatalyst amount, respectively). Since the reaction is quite fast, it compensates the low effectiveness factor, and the average reaction rate (eq 1) is high, the highest of all the systems assayed as previously commented. As for the esterification with 2-ethyl-1-hexanol, the value of *k*_cat_ is the highest (90.13 Mh^–1^ g^–1^) so the reaction is very fast, leading to almost total control of the transport stage and very low values of the effectiveness factor (0.149, 0.123, and 0.107 for 0.5, 0.75, and 1 g biocatalyst amount, respectively), so the average reaction rate is lower than the one obtained with 3,7-dimethyl-1-octanol. Next, using the alcohol 2-butyl-1-octanol, a considerable decrease in the value of *k*_cat_ is observed (8.599 Mh^–1^ g^–1^), which implies a shared control between reaction and transport stages, leading to higher effectiveness factors (0.537, 0.464, and 0.414 for the increasing biocatalyst amounts, respectively). The decrease of the reaction rate is more noticeable than the increase of transport rate, so the global effect is an average reaction rate lower than the previous ones. Finally, for the esterification reaction with 2-hexyl-1-decanol, the value of *k*_cat_ only shows a slight decrease compared to the previous reaction system, but, being this one the alcohol with the highest viscosity and molecular weight as shown in Table 1, the internal diffusional limitations increase, decreasing the effectiveness factor until 0.246, 0.204, and 0.179 for 0.5, 0.75, and 1 g biocatalyst amount. So, for this reaction system, both the reaction stage and transport stage are quite slow, the average reaction rate being the lowest one.

It can be also observed from Table 2 that the effectiveness factor decreases in all cases with the increase of the biocatalyst amount, which is the expected behavior, since the reaction rate increases when *V*_m_ does so, leading to a higher control of the transport stage.

Attending the different carbon-chain length and side-chain structures of the alcohols, it is observed, as expected, that 2-hexyl-1-decanol, the alcohol with both longer carbon-chain length and side-chain structure, presents the worst results. It is known^27^ that a longer chain length leads to more steric hindrance, increased substrate hydrophobicity, and lower diffusivity (higher viscosity), decreasing enzyme–substrate affinity, enzyme binding, and reaction rate. A similar effect is observed with longer side-chain structures. As for the two alcohols with the same chain length, 3,7-dimethyl-1-octanol, with shorter side-chain that is also further from the hydroxyl group, shows better performance than 2-butyl-1-octanol. Finally, the alcohol with the shorter chain length, 2-ethyl-1-hexanol, and lower viscosity shows the higher *k*_cat_ value and higher reaction rate.

### Results for the Temperature Variation

The results obtained for this series for 65, 70, and 75 °C are shown in Figure 2. As can be seen, the experimental conversions match the calculated ones and the conversion order with the different alcohols is the same as in the previous series. Looking at the kinetic parameters shown in Table 3, the explanation for this behavior agrees with the one given in the series of biocatalyst amount variation, since the parameters calculated for 65 and 75 °C show the same tendency than for 70 °C.

**Figure 2:**
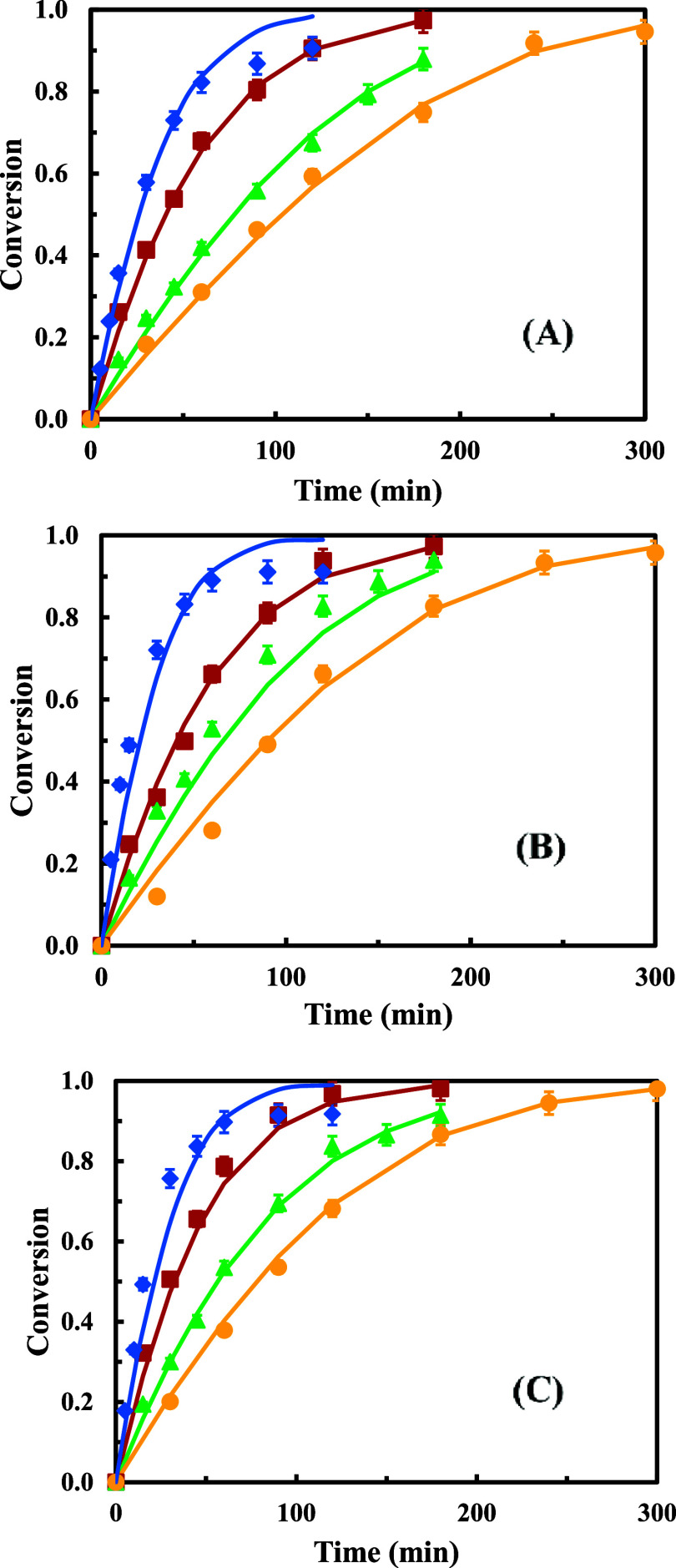
Experimental and calculated conversion values versus time for each reaction system and lauric acid with different alcohols using Novozym 435 as a biocatalyst: (●) 2-hexyl-1-decanol, (▲) 2-butyl-1-octanol, (■) 2-ethyl-1-hexanol, and (⧫) 3,7-dimethyl-1-octanol, (−) calculated. Temperature: (A) 65 °C, (B) 70 °C, and (C) 75 °C. 0.5 g of biocatalyst and a molar ratio acid:alcohol 1:1.

**Table 3:** Kinetic Parameters Obtained for the Series of Temperature Variation

alcohol	*k*_cat_ 65 °C (Mh^–1^ g^–1^)	*k*_cat_ 70 °C (Mh^–1^ g^–1^)	*k*_cat_ 75 °C (Mh^–1^ g^–1^)	η (65 °C)	η (70 °C)	η (75 °C)	error 65 °C	error 70 °C	error 75 °C
**2-ethyl-hexanol**	73.47	90.13	143.41	0.186	0.149	0.137	0.040	0.493	1.000
**3.7-dimethyl-1-octanol**	40.474	49.526	51.856	0.416	0.378	0.373	0.016	0.079	0.154
**2-butyl-1-octanol**	5.702	8.599	17.03	0.577	0.537	0.532	0.125	1.036	0.285
**2-hexyl-1-decanol**	7.126	8.096	9.127	0.259	0.246	0.232	0.614	2.200	0.048

In addition, as expected, the values of *k*_cat_ increase with the increasing temperatures, leading to higher reaction rates and higher control of the internal transport stage, decreasing therefore the effectiveness factors as shown in Table 3.

There is no significant increase in the average reaction rate with the highest temperature. Similar results were obtained by other authors.^28^ Therefore, 70 °C has been selected as the optimum one, on one hand, allowing energy saving compared to 75 °C and on the other hand, compared with the lowest temperature, obtaining higher *k*_cat_ values that lead to some increase in the reaction rate without a significant increase in the internal diffusional limitations.

Figure 3 shows the fitting to the Arrhenius equation. A good fitting is obtained for all reaction systems, except the one with 3,7-dimethyl-1-octanol due to the alcohol evaporation, previously commented, that causes more experimental errors particularly at higher temperatures.

**Figure 3:**
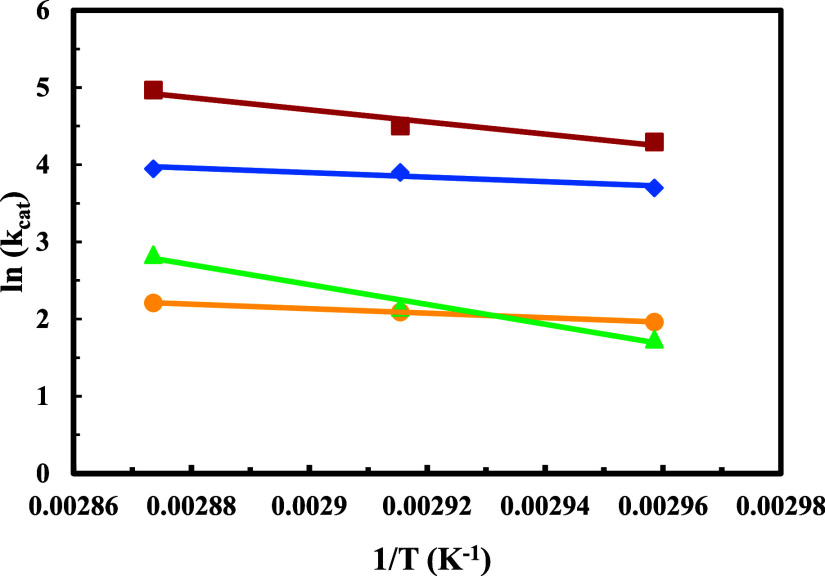
Arrhenius fitting: (●) 2-hexyl-1-decanol, (▲) 2-butyl-1-octanol, (■) 2-ethyl-1-hexanol, and (⧫) 3,7-dimethyl-1-octanol, (−) calculated.

### The Arrhenius Equations for the Reaction Systems Corresponding to Each Alcohol are

2-ethyl hexanol: *y* = 27.482–7851.6*x R* ^2^ = 0.9484.

3,7-dimethyl-1-octanol: *y* = 12.375–2923.6*x R* ^2^ = 0.8888.

2-butyl-1-octanol: *y* = 39.721–1285.3*x R* ^2^ = 0.9773.

2-hexyl-1-decanol *y* = 10.578–2911.3*x R* ^2^ = 0.9999.

### Results for the Molar Ratio Variation

The results from this last series are shown in Figure 4, where for each alcohol corresponding to a different esterification reaction, the experimental and calculated conversion values are depicted for each molar ratio acid:alcohol tested. In addition, the kinetic parameters are listed in Table 4.

**Figure 4:**
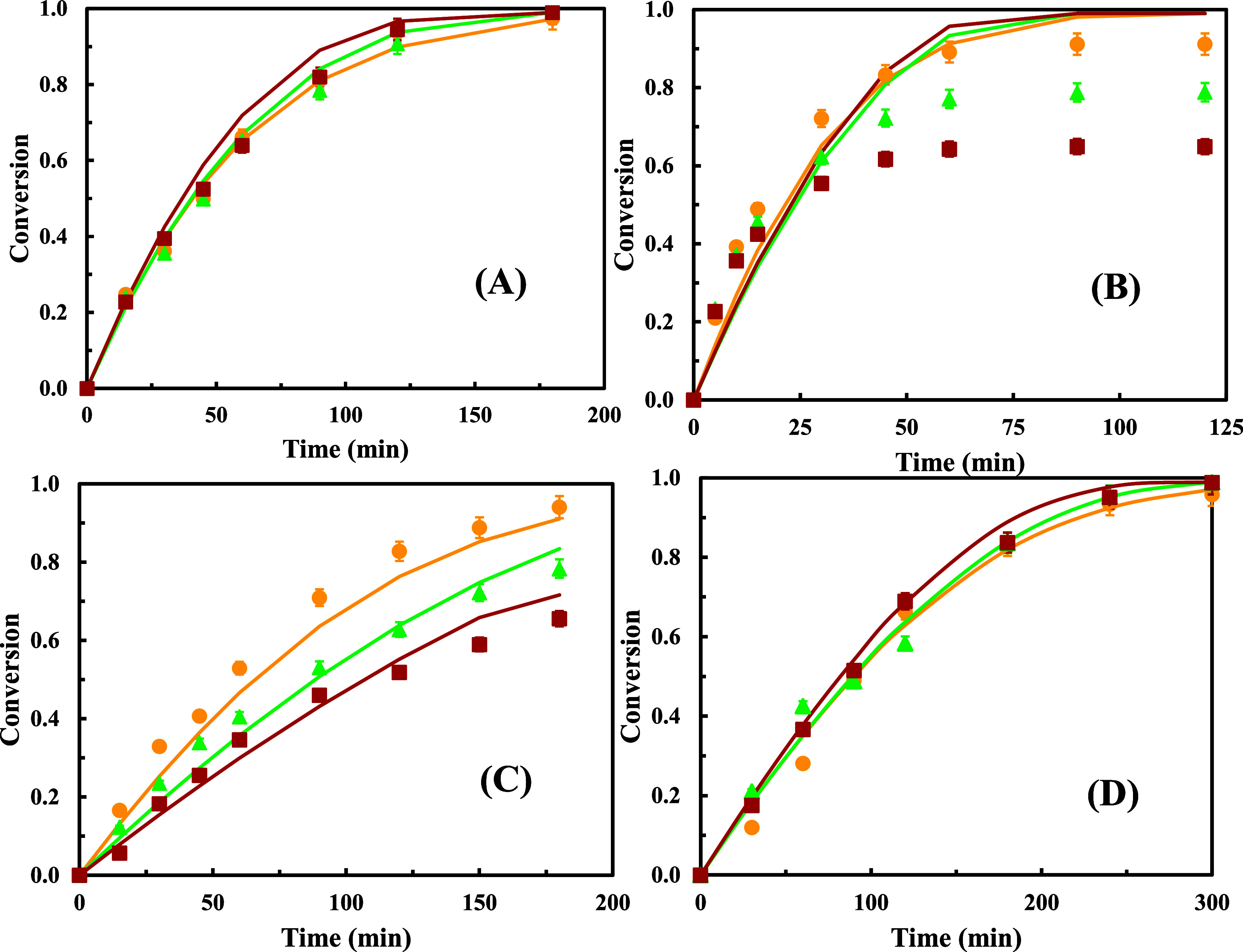
Experimental and calculated conversion values versus time for each reaction system, lauric acid with different alcohols, and molar ratio acid:alcohol using Novozym 435 as a biocatalyst: (●) 1:1, (▲) 1.25:1, (■) 1.5:1, (−) calculated. Alcohol: (A) 2-ethyl-1-hexanol, (B) 3,7-dimethyl-1-octanol, (C) 2-butyl-1-octanol, and (D) 2-hexyl-1-decanol. Temperature of 70 °C and 0.5 g of biocatalyst.

**Table 4:** Kinetic Parameters Obtained for the Series of Molar Ratio Variation

alcohol	*k*_cat_ (Mh^–1^ g^–1^)	Σ*K*_M_ (M)	*K*_MAc_ (M)	*K*_MOl_ (M)	η (1:1)	η (1.25:1)	η (1.50:1)
**2-ethyl-hexanol**	90.13	5.063	2.026	2.957	0.149	0.132	0.129
**3.7-dimethyl-1-octanol**	49.526	2.949	0.678	2.131	0.378	0.272	0.246
**2-butyl-1-octanol**	8.599	2.031	0.723	0.986	0.537	0.325	0.226
**2-hexyl-1-decanol**	8.096	0.935	0.509	0.476	0.246	0.241	0.234

For the reactions with 3,7-dimethyl-1-octanol and 2-butyl-1-octanol, it can be seen from Figure 4B,C that when the molar ratio acid:alcohol increases, there is a decrease in the experimental conversion values as well as in the corresponding effectiveness factors (Table 4). This behavior can be explained considering that an excess of acid leads to an increase of the internal diffusional limitations, decreasing the average rate. It has been previously described^5^ that in these kind of reactions, the first substrate to be attached to the enzyme must be an acyl donor and therefore it must be the lauric acid. So, the presence of an excess of acid could affect the diffusivity of the alcohol inside the pores of the catalyst and therefore increase the internal diffusional limitations. In addition, it has been discussed in previous works that acid excess may affect lipase performance due to a potential acidification of the microaqueous enzyme environment.^16^ Also, as previously mentioned for the esterification with 3,7-dimethyl-1-octanol, the alcohol is more volatile and is progressively lost along the reaction time, becoming more limiting for higher molar ratios acid:alcohol. This also explains the difference between experimental and theoretical conversion values in this case, as previously stated.

For the other reaction systems with 2-ethyl-hexanol and 2-hexyl-1-decanol, the influence of the molar ratio on the obtained conversion values and the effectiveness factors is almost negligible; so, in this case, the excess of acid does not seem to affect the internal transport and neither the average reaction rate. This can be due to the fact that the reaction systems with these two alcohols showed the lowest values of the effectiveness factor with the stoichiometric molar ratio; therefore, the transport stage is already very slow, and adding an excess of acid does not affect it.

Finally, from Table 4, it can be noticed that the sum of the individual Michaelis constants of the acid and the alcohol is very close to the corresponding values of Σ*K*_M_ shown in Table 2, which reinforces the validity of the proposed kinetic model. The main purpose of this series is, precisely, obtaining these individual values of the Michaelis constants from eq 2 that can only be used when the molar ratio is different from the stoichiometric one (which is the one leading to the best experimental results).

## Conclusions

From the comparison of the four esterification systems tested, it can be concluded that the one with the alcohol 3,7-dimethyl-1-octanol is the fastest in terms of the average rate considering both the reaction rate and the internal diffusional limitations. This alcohol, however, is quite volatile, so at long reaction times, it becomes limiting, and there is a decrease in conversion values. Higher molar ratios of alcohol:acid should be tested to improve this behavior. The system with 2-ethyl-1-hexanol presents the highest values of *k*_cat_ and reaction rate and, therefore, a main control of the internal transport and the lowest values of the effectiveness factors, although the average rate is quite high. On the contrary, the esterification with 2-hexyl-1-decanol shows the worst results due to the high molecular weight and viscosity of the alcohol and longer chain length and side chain, having the lowest value of *k*_cat_; so, both the reaction and the transport stages are quite slow.

In the two systems that present high internal diffusional limitations with the stoichiometric molar ratio acid:alcohol, the effect of increasing this ratio is negligible, while for the reaction systems using 3,7-dimethyl-1-octanol and 2-butyl-1-octanol, the excess of acid has a negative effect, increasing the internal diffusional limitations and thus decreasing the overall rate of the process.

From the different reaction parameters tested, the selected ones have been biocatalyst amount 0.5 g, temperature 70 °C, and molar ratio acid:alcohol 1:1 (the stoichiometric one). Higher values of these parameters lead to higher reaction costs and higher internal diffusional limitations. Under these optimum conditions, the obtained branched esters can be useful in the biolubricant sector, although further characterization is needed in this sense.

The proposed kinetic model has been shown to be valid for all the esterification systems (except for the one with 3,7-dimethyl-1-octanol at long reaction times due to the high alcohol volatility), obtaining the kinetic parameters with the Excel Solver tool and a very good fitting between the experimental and calculated conversion values. The model validation for systems of alcohols with different chain length and side chain allows its use for simulation, optimization, and scale-up of these types of enzymatic processes.

## Abbreviations

Ac: acid
Ol: alcohol
*C*_Ac_: acid concentration (M)
*C*_Ol_: alcohol concentration (M)
*C*_Ac0_: initial acid concentration (M)
*C*_Ol0_: initial alcohol concentration (M)
*V*_m_: maximum reaction rate of enzyme (M h^–1^)
*k*_cat_: enzyme catalytic constant (M h^–1^ g^–1^)
*K*_Mol_: Michaelis constant of alcohol (M)
*K*_Mac_: Michaelis constant of acid (M)
Σ*K*_M_: sum of Michaelis constants (M)
*r*: reaction rate (M h^–1^)
*t*: experimental reaction time (h)
*t*_calc_: calculated reaction time (h)
*X*: experimental conversion (dimensionless)
*X*_calc_: calculated conversion (dimensionless)
η: enzymatic reaction effectiveness factor (dimensionless).
